# Functional Relationship between Sucrose and a Cariogenic Biofilm Formation

**DOI:** 10.1371/journal.pone.0157184

**Published:** 2016-06-08

**Authors:** Jian-Na Cai, Ji-Eun Jung, Minh-Huy Dang, Mi-Ah Kim, Ho-Keun Yi, Jae-Gyu Jeon

**Affiliations:** 1 Department of Preventive Dentistry, School of Dentistry, Institute of Oral Bioscience, Chonbuk National University, Jeonju, Republic of Korea; 2 Department of Oral Biochemistry, School of Dentistry, Institute of Oral Bioscience, Chonbuk National University, Jeonju, Republic of Korea; Laurentian, CANADA

## Abstract

Sucrose is an important dietary factor in cariogenic biofilm formation and subsequent initiation of dental caries. This study investigated the functional relationships between sucrose concentration and *Streptococcus mutans* adherence and biofilm formation. Changes in morphological characteristics of the biofilms with increasing sucrose concentration were also evaluated. *S*. *mutans* biofilms were formed on saliva-coated hydroxyapatite discs in culture medium containing 0, 0.05, 0.1, 0.5, 1, 2, 5, 10, 20, or 40% (w/v) sucrose. The adherence (in 4-hour biofilms) and biofilm composition (in 46-hour biofilms) of the biofilms were analyzed using microbiological, biochemical, laser scanning confocal fluorescence microscopic, and scanning electron microscopic methods. To determine the relationships, 2^nd^ order polynomial curve fitting was performed. In this study, the influence of sucrose on bacterial adhesion, biofilm composition (dry weight, bacterial counts, and water-insoluble extracellular polysaccharide (EPS) content), and acidogenicity followed a 2^nd^ order polynomial curve with concentration dependence, and the maximum effective concentrations (MECs) of sucrose ranged from 0.45 to 2.4%. The bacterial and EPS bio-volume and thickness in the biofilms also gradually increased and then decreased as sucrose concentration increased. Furthermore, the size and shape of the micro-colonies of the biofilms depended on the sucrose concentration. Around the MECs, the micro-colonies were bigger and more homogeneous than those at 0 and 40%, and were surrounded by enough EPSs to support their structure. These results suggest that the relationship between sucrose concentration and cariogenic biofilm formation in the oral cavity could be described by a functional relationship.

## Introduction

Dental caries, a biofilm-related disease, is associated with the presence of cariogenic bacteria and high consumption of dietary carbohydrates [1, 2]. Among dietary carbohydrates, sucrose can cause major biochemical and physiological changes during dental biofilm formation and is considered one of the most cariogenic carbohydrates [1, 3]. Sucrose fermentation by oral bacteria can rapidly reduce the pH in dental biofilms, which results in a shift in the balance of resident plaque microflora to become more cariogenic [4]. Sucrose also serves as a substrate for the synthesis of polysaccharides in dental biofilms, especially extracellular polysaccharides (EPSs) [5]. In addition, recent studies have demonstrated that sucrose can reduce the concentrations of calcium (Ca), inorganic phosphorus (P_i_), and fluoride (F) in the dental biofilms; these are critical ions involved in the demineralization and remineralization of enamel and dentin in the oral environment [6, 7].

Among cariogenic bacteria, *Streptococcus mutans* is generally regarded as a primary etiologic agent of dental caries [8, 9]. This bacterium can produce large amounts of acid and survive in a low pH environment. Furthermore, *S*. *mutans* can utilize dietary sucrose to synthesize EPSs, which are mostly glucans synthesized by glucosyltransferases (GTFs) [10–12]. The bacterium produces at least three GTFs (GTFB, GTFC, and GTFD), and synthesizes a mixture of α(1→3)-linked insoluble and α(1→6)-linked soluble EPSs [13, 14]. The EPSs, especially water-insoluble EPS, can promote selective adherence and accumulation of large numbers of cariogenic streptococci on the tooth surface, which contribute to the cariogenic biofilm formation [15, 16].

Many epidemiological and experimental studies have been performed to reveal the relationship between sucrose and dental caries development [17–20]. Recent studies have demonstrated that the cariogenicity of sucrose is related to the concentration and frequency of exposure [21, 22]. Furthermore, several *in vivo* studies have shown that there is a strong relationship between sucrose concentration in the diet and the incidence of smooth surface and fissure caries [23], and that sucrose concentration also can influence pH in dental plaque *in vivo* [24]. However, few studies have been performed to investigate the precise relationship between sucrose concentration and dental caries development. Furthermore, little has been reported on the functional relationship between sucrose concentration and cariogenic biofilm formation, in particular for *S*. *mutans*.

Therefore, the aim of this study was to evaluate the functional relationships between sucrose concentration and *S*. *mutans* adherence and biofilm formation. We also investigated the changes in morphological characteristics of the biofilms according to sucrose concentration.

## Materials and Methods

### *S*. *mutans* biofilm preparation and sucrose

*S*. *mutans* UA159 (ATCC 700610; serotype c) biofilms were formed on saliva-coated hydroxyapatite (sHA) discs (2.93 cm^2^; Clarkson Chromatography Products, Inc., South Williamsport, PA, USA) placed in a vertical position in 24-well plates, as detailed elsewhere [25]. Briefly, sHA discs were generated by incubation with filter-sterilized (0.22-μm low protein-binding filter) human whole saliva for 1 h at 37°C. For biofilm formation, the sHA discs were transferred to a 24-well plate containing brain heart infusion (BHI; Difco, Detroit, MI, USA) broth with 0, 0.05, 0.1, 0.5, 1, 2, 5, 10, 20, or 40% (w/v) sucrose and *S*. *mutans* UA159 (2–5×10^6^ colony-forming unit (CFU)/ml). The biofilms were grown at 37°C with 5% CO_2_ for 4 h or 46 h. For 46-h biofilm formation, the biofilms were grown undisturbed for 22 h to allow initial biofilm growth and then the culture medium (0, 0.05, 0.1, 0.5, 1, 2, 5, 10, 20, or 40% sucrose BHI broth) was changed twice, at 22 and 31 h.

### Microbiological and biochemical studies

#### Bacterial adhesion analysis

To determine the change in *S*. *mutans* adhesion in the presence of different concentrations of sucrose, the 4-h biofilms were transferred into 5 ml of 0.89% NaCl and sonicated in an ultrasonic bath (Power sonic 410; Hwashin Technology Co., Seoul, Korea) for 10 min to detach the biofilms. The biofilm solution was resonicated at 7 W for 30 s (VCX 130PB; Sonics and Materials Inc., Newtown, CT, USA). An aliquot (0.1 ml) of the dispersed solution was serially diluted and plated on BHI agar plates for determination of CFU as detailed elsewhere [26].

#### Biofilm formation analysis

The 46-h biofilms were analyzed to determine the change in *S*. *mutans* biofilm formation according to the concentration of sucrose. The 46-h biofilms were detached and sonicated to analyze CFU count as described above. The dry weight and the amount of water-insoluble EPSs were determined as described elsewhere [26]. Briefly, the remaining solution (4.9 ml) was centrifuged (3000 g) for 20 min at 4°C. The biofilm pellet was re-suspended and washed twice in an equivalent volume of water. The washed biofilm pellet was lyophilized and weighed to determine the biofilm biomass. In addition, the water-insoluble EPSs were extracted from the dry pellet using 1 N sodium hydroxide before determination of the polysaccharide amount using a phenol-sulfuric acid assay. The final pH values of the old culture media were also determined during the experimental period using a glass electrode (Beckman Coulter Inc., Brea, CA, USA) and the H^+^ production rate (μM/h) was calculated by the pH values (from 22 to 31 h) to investigate the change in acidogenicity of the biofilms.

### Confocal laser scanning microscopy

Confocal laser scanning microscopy (CLSM) was performed as described by Jeon et al. [25] to confirm the results of microbiological and biochemical studies. The concentrations of sucrose tested in the CLSM study were 0, 1, 10, and 40% (w/v). To determine the change in *S*. *mutans* adhesion and biofilm formation, 1 μM of Alexa Fluor® 647-labeled dextran conjugate (10,000 MW; absorbance/fluorescence emission maxima 647/668 nm; Molecular Probes Inc., Eugene, OR, USA) was added to 0, 1, 10, or 40% sucrose BHI broth with *S*. *mutans* UA159 (2–5 × 10^6^ CFU/ml) at 0, 22, and 31 h. The fluorescence-labeled dextran serves as a primer for GTFs and can be simultaneously incorporated during the extracellular polysaccharide matrix synthesis over the course of the biofilm development, but does not stain the bacterial cells at concentrations used in this study [25]. After 4 or 46 h, the bacterial cells in the biofilms were labeled by incubation with 2.5 μM SYTO 9 green fluorescent nucleic acid stain (480/500 nm; Molecular Probes Inc.) for 30 min. CLSM imaging of the biofilms was performed using a LSM 510 META (Carl Zeiss, Jena, Germany) microscope equipped with argon ion and helium-neon lasers. Four independent experiments were performed and five image stacks per experiment were collected (n = 20). The bio-volume (μm^3^/μm^2^), mean thickness (μm), roughness coefficient, and surface to volume ratio (μm^2^/μm^3^) of bacterial micro-colonies and EPSs were quantified from the confocal stacks using COMSTAT [27]. The bio-volume is defined as the volume of the biomass (μm^3^) divided by the surface area of the substratum (HA discs) (μm^2^). The roughness coefficient, which provides a measure of how much the thickness of the biofilm varies, is calculated from the thickness distribution of the biofilm. The surface to volume ratio is the surface area divided by the bio-volume. The roughness coefficient and surface to volume ratio reflect the degree of biofilm heterogeneity and nutrient exposure, respectively [27]. The three-dimensional architecture of the biofilms was visualized using Imaris 8.0.2 (Bitplane, Zurich, Switzerland).

### Scanning electron microscopy

The scanning electron microscopy (SEM) study was performed as detailed elsewhere [28]. Briefly, 46-h biofilms grown in 0, 1, 10, or 40% sucrose BHI broth were rinsed three times in 0.1 M cacodylate buffer and prefixed with 3% glutaraldehyde solution for 1 h followed by post-fixation with a 1% osmium tetroxide solution for 1 h. The biofilms were then dehydrated in a graded series of ethanol (30–100%) and infiltrated with nitrogen gas immediately before sputter coating with gold–palladium. The biofilm samples were analyzed by SEM (JSM-5900, Jeol, Japan).

### Statistical analysis

To determine the relationships between sucrose concentration and *S*. *mutans* adherence or biofilm formation, 2^nd^ order polynomial fitting for sucrose concentration versus CFU count, dry weight, amount of water-insoluble EPSs, or final pH value was performed using a polynomial regression analysis program (Origin 7.0; Microcal, Inc., Northampton, MA, USA). The determination coefficients (R^2^) of each fitted line were also calculated.

All experiments were performed in duplicate, and at least four different experiments were conducted. The data are presented as mean ± standard deviation. Intergroup differences were estimated using one-way analysis of variance, followed by a post hoc multiple comparison (Tukey) test to compare the multiple means. Values were considered statistically significant when the *P* value was < 0.05.

## Results

### Relationship between sucrose concentration and *S*. *mutans* adhesion

**Fig 1:**
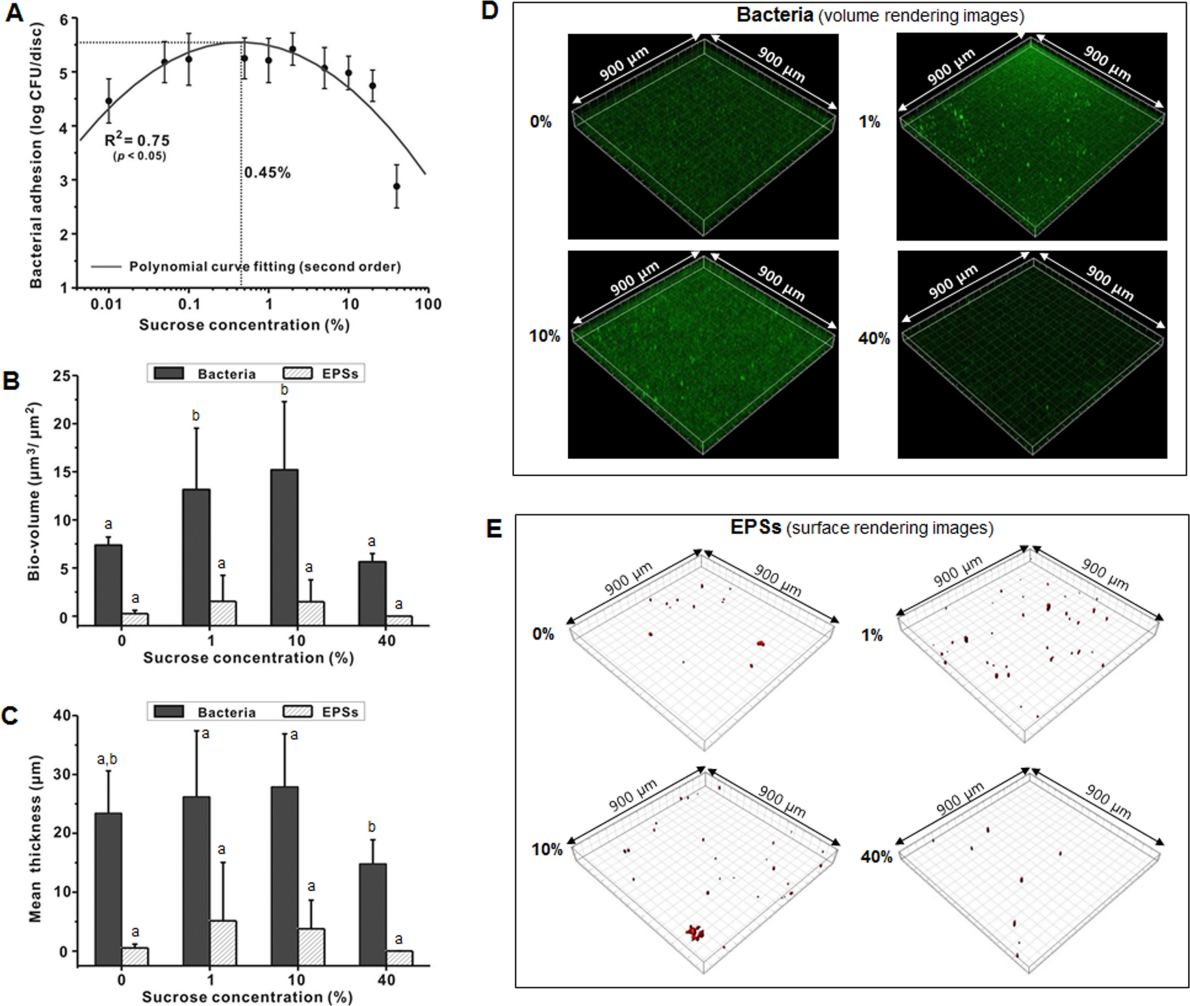
Relationship between sucrose concentration and *S*. *mutans* adhesion at 4 h. (**A**) Bacterial adhesion. (**B**) Bio-volume. (**C**) Mean thickness. (**D**) Representative confocal images of bacteria in the biofilms. (**E**) Representative confocal images of EPS in the biofilms. Values followed by the same letters are not significantly different from each other (*P* > 0.05).

As shown in Fig 1A, *S*. *mutans* adhesion during 4-h biofilm formation gradually increased and then decreased as the sucrose concentration increased. Generally, the change in *S*. *mutans* adhesion to sHA discs followed a 2^nd^ order polynomial curve with sucrose concentration dependence. The R^2^ value of the polynomial curve was 0.75 (*P* < 0.05). The maximum effective concentration (MEC) of sucrose for bacterial adhesion from the polynomial curve was 0.45%. In the CLSM study, the bacterial bio-volume of the 4-h biofilms also gradually increased and then decreased as the sucrose concentration increased (Fig 1B). The bacterial bio-volume at 1 and 10% was at least 1.5 times higher than that at 0 and 40% (*P* < 0.05). However, the mean bacterial thickness did not change with increasing sucrose concentration (Fig 1C). The EPS bio-volume and mean thickness also showed no change according to sucrose concentration, except at 40% (Fig 1B and 1C). Representative bacterial and EPS CLSM images are shown in Fig 1D and 1E, respectively, and show that bacterial micro-colonies were more prominent at 1 and 10% than those at 0 and 40%, but EPSs were barely detected at any of the concentrations tested.

### Relationship between sucrose concentration and *S*. *mutans* biofilm formation

#### Relationship in microbiological and biochemical studies

**Fig 2:**
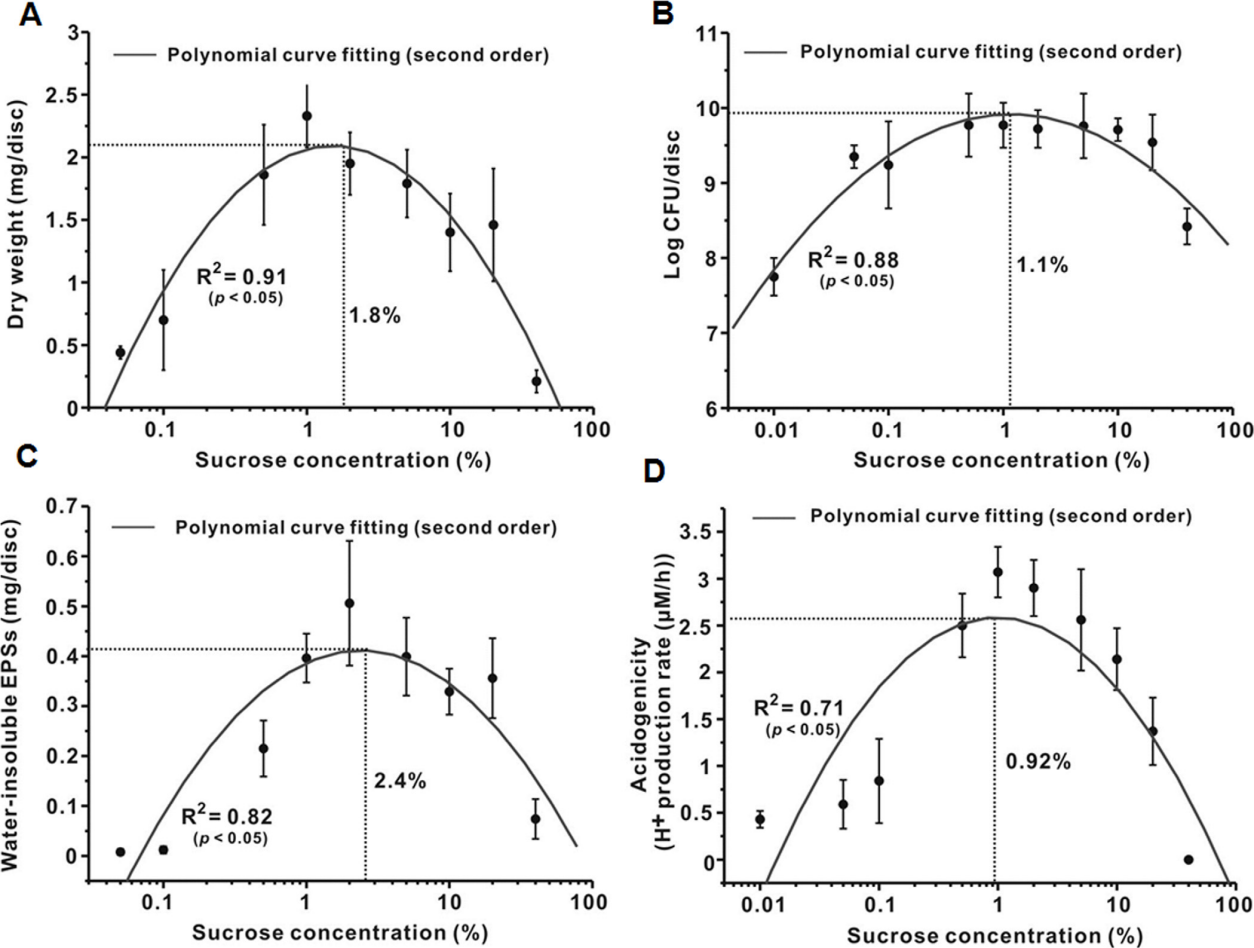
Relationship between sucrose concentration and *S*. *mutans* biofilm formation. (**A**) Dry weight. (**B**) Bacterial viability. (**C**) Water-insoluble EPSs. (**D**) Acidogenicity. In A and C, the dry weight and amount of water-insoluble EPSs at 0% were not detected. In B and D, since x coordinates are plotted on a logarithmic scale, and since the log of 0 is undefined, we approximated 0 with an x coordinate of 0.01.

As shown in Fig 2A, the dry weight of the 46-h biofilms gradually increased and then decreased as sucrose concentration increased, which followed a 2^nd^ order polynomial curve with sucrose concentration dependence. The R^2^ value of the polynomial curve was 0.91 (*P* < 0.05). The MEC of sucrose for dry weight in the polynomial curve was 1.8%. The change in CFU counts, water-insoluble EPS amount, and acidogenicity of the 46-h biofilms also followed a 2^nd^ order polynomial curve with sucrose concentration dependence (Fig 2B–2D); the R^2^ values were 0.88, 0.82, and 0.71 (*P* < 0.05), respectively. The MECs ranged from 0.92 to 2.4%. Generally, the results showed that the change in the composition and virulence of *S*. *mutans* biofilms followed a 2^nd^ order polynomial curve with sucrose concentration dependence.

#### Biofilm change in CLSM and SEM study

**Fig 3:**
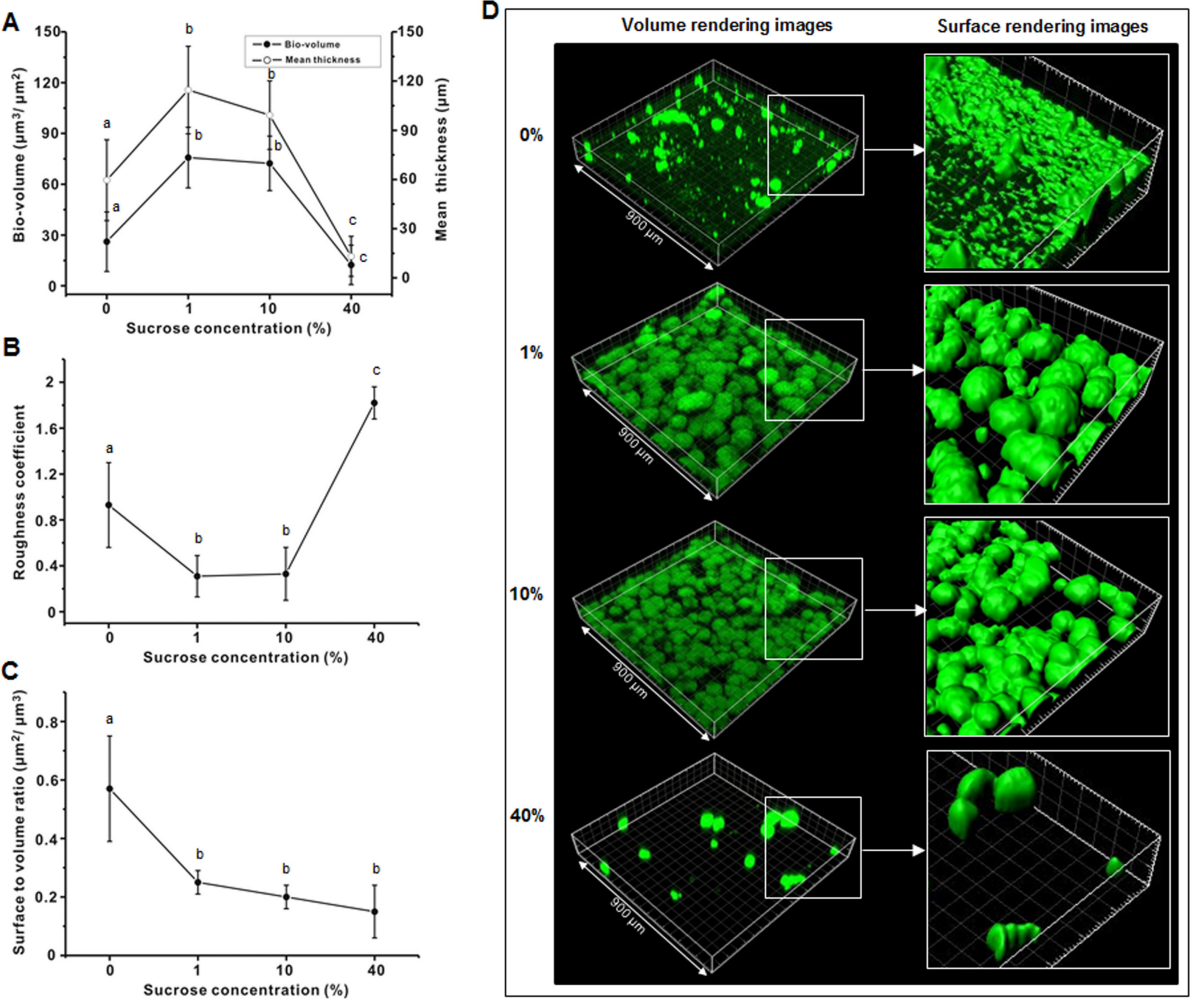
Effect of sucrose concentration on bacteria (**A**) bio-volume and mean thickness (**B**) roughness coefficient, and (**C**) surface to volume ratio of 46-h *S*. *mutans* biofilm. (**D**) Representative 3-D images (isosurface rendering) of bacterial cells. Values followed by the same letters are not significantly different from each other (*P* > 0.05).

In the present study, the bio-volume, mean thickness, roughness coefficient, and surface to volume ratio of the 46-h biofilms were analyzed to evaluate changes in the biofilm structure with increasing sucrose concentration. As shown in Fig 3A, the bio-volume and mean thickness of the bacterial micro-colonies of the 46-h biofilms initially increased gradually and then decreased as the sucrose concentration increased. The bacterial bio-volume and mean thickness at 1 and 10% were at least 1.5 times higher than those at 0 and 40% (*P* < 0.05). However, the roughness coefficient of the bacterial micro-colonies gradually decreased and then increased as sucrose concentration increased (Fig 3B); the roughness coefficient at 1 and 10% was at least 2.5 times lower than that at 0 and 40%. The surface to volume ratio of the bacterial micro-colonies at 0% was higher than that at 1, 10, and 40% (Fig 3C). Fig 3D shows representative bacterial images from the CLSM study, in which the bacterial micro-colonies at 1 and 10% were bigger, more spherical, and more aggregated than that at 0%, whereas there were only scattered large micro-colonies at 40% (Fig 3D).

**Fig 4:**
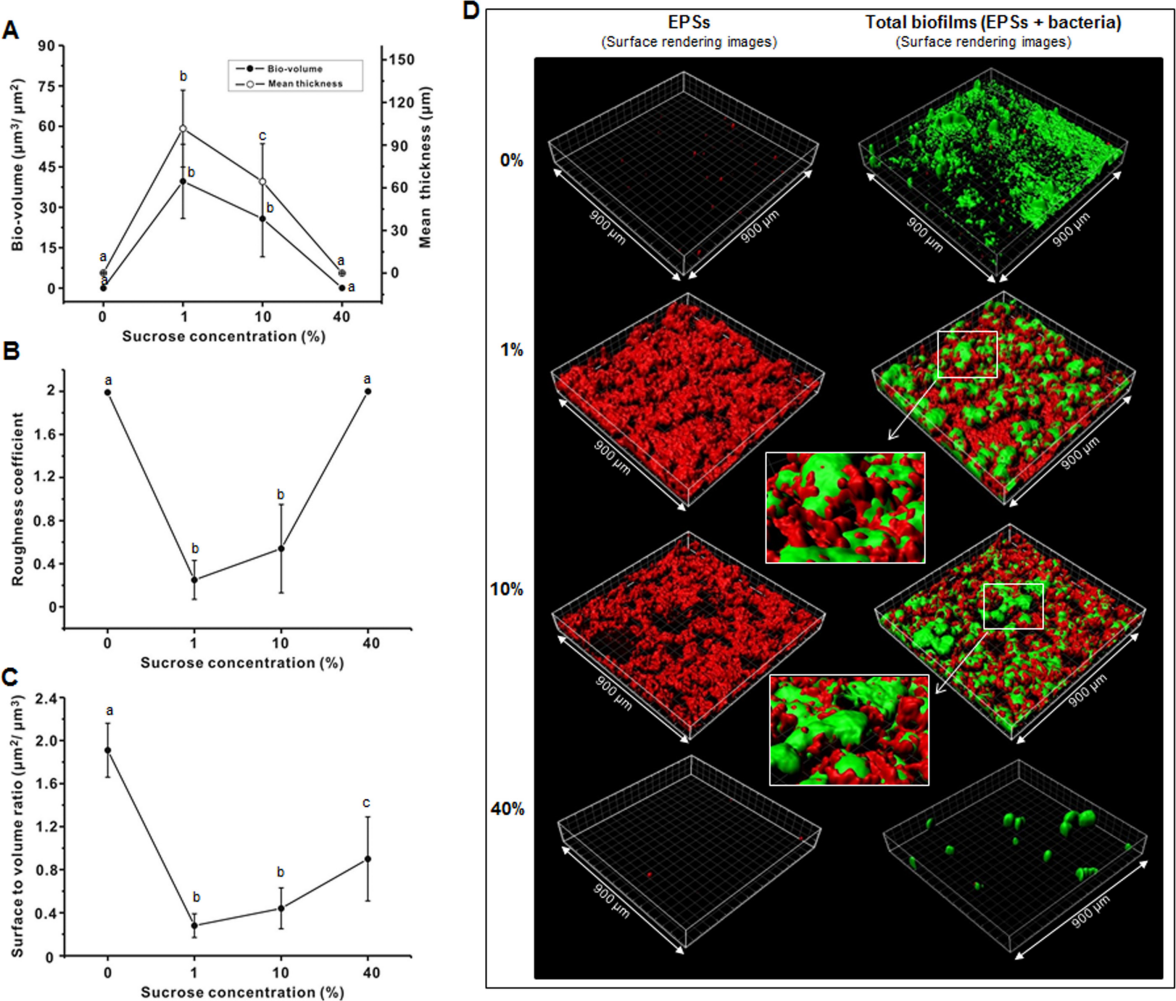
Effect of sucrose concentration on EPS (**A**) bio-volume and mean thickness (**B**) roughness coefficient, and (**C**) surface to volume ratio of 46-h *S*. *mutans* biofilm. (**D**) Representative 3-D images (isosurface rendering) of bacterial cells (green) and EPS (red). Values followed by the same letters are not significantly different from each other (*P* > 0.05).

The bio-volume and mean thickness of the EPSs in the 46-h biofilms also gradually increased and then decreased as sucrose concentration increased (Fig 4A). The bio-volume and mean thickness of the EPSs were minimal at 0 and 40%, but EPSs were strongly formed at 1 and 10%. Fig 4B and 4C show the roughness coefficient and surface to volume ratio of the EPSs, which showed an opposite pattern to that observed for bio-volume and mean thickness. Fig 4D shows representative EPS and total biofilm (EPSs + bacteria) surface rendering images, in which the EPSs were hardly detected at 0 and 40% and the bacterial micro-colonies were not covered by EPSs at these concentrations.

**Fig 5:**
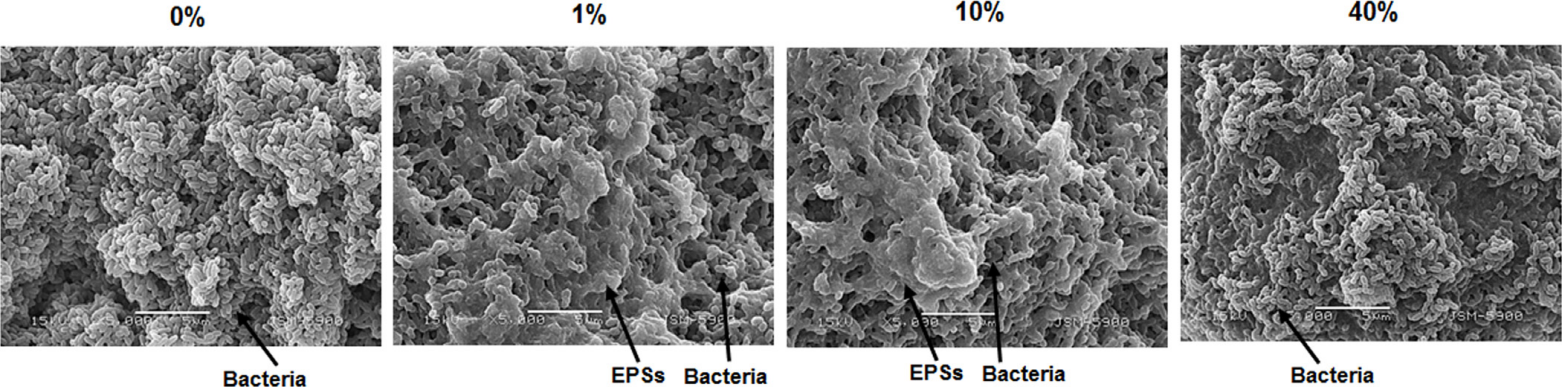
Representative SEM images (5000×) of 46-h *S*. *mutans* biofilms in different concentrations of sucrose (0, 1, 10, 40%).

In addition, Fig 5 shows representative SEM images (5,000×) of the 46-h biofilms in different concentrations of sucrose. It was apparent that the biofilms at 1 and 10% exhibited a larger amount of EPSs covering the micro-colonies than those at 0 or 40%, which was consistent with the results of the CLSM study (Figs 3 and 4).

## Discussion

Biofilm formation is a complex process that is affected by many factors such as growth environment, nutrition, bacterial vitality, and surface characteristics [29, 30]. Sucrose, an important substrate for dental biofilm formation, has been studied in a series of epidemiologic and experimental studies, which confirmed that sucrose can cause major biochemical and physiological changes during the process of cariogenic biofilm formation, and that, in turn, enhance its caries-inducing properties [17–19, 31, 32]. However, limited studies have been performed to evaluate the effects of sucrose level on cariogenic biofilm formation. Therefore, in the present study, we determined the relationship between sucrose concentration and *S*. *mutans* adherence and biofilm formation. Although the biofilm mode used in the present study could provide significant benefits of establishing the reproducibility of data and reducing variance [28], additional studies are required to confirm the relationships between sucrose concentration and its influence on cariogenic biofilms *in vivo* since *S*. *mutans* does not occur in a monoculture *in vivo* and the environmental conditions in the present study differ from those in the oral cavity.

In the present study, 2^nd^ order polynomial fitting was performed to determine the relationships between sucrose concentration and *S*. *mutans* adherence or biofilm formation. The coefficient of determination (R^2^) of the polynomial curves in the present study ranged from 0.64 to 0.91 (*P* < 0.05). This result indicates that the polynomial curves appropriately describe *S*. *mutans* adherence or biofilm formation in relation to sucrose concentration, and that 64–91% of the variation in *S*. *mutans* adherence or biofilm formation can be explained by variation in sucrose concentration.

Adhesion of bacteria to a surface is a prerequisite for biofilm formation, and contributes to both biofilm development and maturation [29]. In the present study, adhesion of *S*. *mutans* to sHA discs followed a 2^nd^ order polynomial curve with sucrose concentration dependence (MEC: 0.45%) (Fig 1A). Our data on the bacterial bio-volume during 4-h incubation further confirmed this pattern (Fig 1B–1D). These findings suggest that increasing sucrose levels increase *S*. *mutans* adhesion up to a certain concentration (turning concentration), after which bacterial adhesion decreases as sucrose concentration increases. In the present study, the turning concentration for *S*. *mutans* adhesion was 0.45% (Fig 1A). Although the mechanism by which *S*. *mutans* adhesion was reduced at high concentration of sucrose was not revealed in the present study, the reduction may be due to the inhibitory effect on bacterial growth in a planktonic state. According to a previous study, the growth of *Listeria monocytogenes*, a gram-positive bacterium, was also strongly affected at 20–60% sucrose [33], suggesting that the total number of gram-positive bacteria that can adhere to a surface might be reduced at high concentrations of sucrose. In addition, the EPS bio-volume and thickness that formed during the bacterial adhesion stage were not affected by sucrose concentration in the present study (Fig 1B and 1C). This result may reflect the amount of EPSs synthesized during the experiment. As shown in Fig 1B and 1E, the EPS bio-volume formation during 4-h incubation was too low to allow precise comparisons using CLSM images.

After adhesion to the surface, bacterial cells will accumulate and subsequently form micro-colonies through an EPS-mediated process, leading to biofilm formation [13]. As shown in Fig 2, sucrose influences the dry weight, CFU counts, water-insoluble EPSs, and acidogenicity of the 46-h biofilms in a 2^nd^ order polynomial curve with concentration dependence. These findings suggest that sucrose can increase the accumulation and virulence of *S*. *mutans* biofilms up to a certain concentration (turning concentration), but the accumulation and virulence decrease as sucrose concentration further increases. In addition, in the present study, the maximum effective concentrations of sucrose for *S*. *mutans* biofilm formation ranged from 0.45 to 2.4% (Figs 1 and 2). Interestingly, however, a previous study reported that the threshold of sucrose concentration for the formation of a cariogenic biofilm is 5%, in which 1–40% sucrose was treated 8 times (5 min/time) per day for 14 days [21]. The difference between the present and previous study can be attributed to differences in treatment duration, suggesting that the effect of sucrose on cariogenic biofilm formation is closely related to both concentration and treatment duration. Therefore, further studies will be needed to reveal the precise relationship between sucrose treatment duration and cariogenic biofilm formation.

In the present study, the appearance of the micro-colonies of the 46-h biofilms was also closely related to sucrose concentration (Fig 3D). As shown in Fig 3B, the roughness coefficient of the micro-colonies at 1 and 10% was at least 2.5 times lower than that at 0 and 40%, indicating that the micro-colonies at 1 and 10% were more homogeneous than that at 0 and 40%. However, interestingly, the surface to volume ratio at 40% was lower than that at 0% (Fig 3B and 3C), meaning that the exposure of the micro-colonies to nutrient flow was lower at 40% than at 0%. This result suggests that the exposure to nutrient flow at 40% was very limited even though the sucrose concentration was the highest tested. In general, although our findings confirm the crucial role of sucrose in micro-colony development and in maintaining the three-dimensional structure of biofilms [13], they suggest an adverse effect on homogeneity and exposure to nutrient flow of biofilm micro-colonies at very high sucrose concentrations.

It is well documented that EPSs contribute to the bulk and physical integrity and stability of the biofilm matrix [15, 34]. The change in EPSs also closely related to sucrose concentration (Fig 4). In the 46-h biofilms, the bio-volume and mean thickness of the EPSs at 1 and 10% were significantly higher than those at 0 and 40% (*P* < 0.05) (Fig 4A), confirming the data from the biochemical study and SEM images (Figs 2C and 5). Furthermore, the EPSs produced at 1 and 10% could cover the micro-colonies (Fig 4D). This result suggests that the micro-environment of the biofilms at 1 and 10% is different from that at 0 and 40% since the EPSs surrounding the micro-colonies may create chemical gradients due to differential diffusion of nutrients and metabolic products throughout the biofilm matrix [13].

Collectively, the results of the present study revealed that the effect of sucrose on *S*. *mutans* adhesion and biofilm formation followed a 2^nd^ order polynomial curve with concentration dependence and the turning concentration ranged from 0.45 to 2.4%. These results can provide a fundamental basis for a more precise study on cariogenic biofilm development in relation to sucrose concentration. However, additional studies are required to confirm the statistical relationships between sucrose concentration and its influence on cariogenic biofilms *in vivo*.

## References

[1] Bowen WH. Do we need to be concerned about dental caries in the coming millennium? Crit Rev Oral Biol Med. 2002;13(2): 126–131. 12097355 10.1177/154411130201300203

[2] Marsh PD. Are dental diseases examples of ecological catastrophes? Microbiology. 2003;149(2): 279–294. 12624191 10.1099/mic.0.26082-0

[3] Paes Leme AF, Koo H, Bellato CM, Bedi G, Cury JA. The role of sucrose in cariogenic dental biofilm formation–new insight. J Dent Res. 2006;85(10): 878–887. 16998125 10.1177/154405910608501002 PMC2257872

[4] Marsh PD. Controlling the oral biofilm with antimicrobials. J Dent. 2010;38(Suppl 1): S11–S15. 10.1016/S0300-5712(10)70005-1 20621238

[5] Kreth J, Zhu L, Merritt J, Shi W, Qi F. Role of sucrose in the fitness of *Streptococcus mutans*. Oral Microbiol Immunol. 2008;23(3): 213–219. 10.1111/j.1399-302X.2007.00413.x 18402607

[6] Tenuta LM, Del Bel Cury AA, Bortolin MC, Vogel GL, Cury JA. Ca, Pi, and F in the fluid of biofilm formed under sucrose. J Dent Res. 2006;85(9): 834–838. 16931867 10.1177/154405910608500911

[7] Paes Leme AF, Dalcico R, Tabchoury CP, Del Bel Cury AA, Rosalen PL, Cury JA. In situ effect of frequent sucrose exposure on enamel demineralization and on plaque composition after APF application and F dentifrice use. J Dent Res. 2004;83(1): 71–75. 14691117 10.1177/154405910408300114

[8] Loesche WJ. Role of *Streptococcus mutans* in human dental decay. Microbiol Rev. 1986;50(4): 353–380 3540569 10.1128/mr.50.4.353-380.1986 PMC373078

[9] Mogen AB, Chen F, Ahn SJ, Burne RA, Wang D, Rice KC. Pluronics Formulated Farnesol Promotes Efficient Killing and Demonstrates Novel Interactions with Streptococcus mutans Biofilms. PLoS One. 2015;10(7): e0133886 10.1371/journal.pone.0133886 26222384 PMC4519314

[10] Bowen WH, Koo H. Biology of *Streptococcus mutans*-derived glucosyltransferases: role in extracellular matrix formation of cariogenic biofilms. Caries Res. 2011; 45(1): 69–86. 10.1159/000324598 21346355 PMC3068567

[11] Sendamangalam V, Choi OK, Kim D, Seo Y. The anti-biofouling effect of polyphenols against *Streptococcus mutans*. Biofouling. 2011;27(1): 13–19. 10.1080/08927014.2010.535897 21104475

[12] de Sousa DL, Lima RA, Zanin IC, Klein MI, Janal MN, Duarte S. Effect of Twice-Daily Blue Light Treatment on Matrix-Rich Biofilm Development. PLoS One. 2015;10(7): e0131941 10.1371/journal.pone.0131941 26230333 PMC4521953

[13] Koo H, Xiao J, Klein MI, Jeon JG. Exopolysaccharides produced by *Streptococcus mutans* glucosyltransferases modulate the establishment of microcolonies within multispecies biofilms. J Bacteriol. 2010;192(12): 3024–3032. 10.1128/JB.01649-09 20233920 PMC2901689

[14] Kim D, Hwang G, Liu Y, Wang Y, Singh AP, Vorsa N, et al Cranberry Flavonoids Modulate Cariogenic Properties of Mixed-Species Biofilm through Exopolysaccharides-Matrix Disruption. PLoS One. 2015;10(12): e0145844 10.1371/journal.pone.0145844 26713438 PMC4699891

[15] Schilling KM, Bowen WH. Glucans synthesized in situ in experimental salivary pellicle function as specific binding sites for *Streptococcus mutans*. Infect Immun. 1992;60(1): 284–295. 1530843 10.1128/iai.60.1.284-295.1992 PMC257534

[16] Vacca-Smith AM, Bowen WH. Binding properties of streptococcal glucosyltransferases for hydroxyapatite, saliva-coated hydroxyapatite, and bacterial surfaces. Arch Oral Biol. 1998;43(2): 103–110. 9602288 10.1016/s0003-9969(97)00111-8

[17] Cury JA, Rebello MA, Del Bel Cury AA. In situ relationship between sucrose exposure and the composition of dental plaque. Caries Res. 1997;31(5): 356–360. 9286518 10.1159/000262418

[18] Cury JA, Rebelo MA, Del Bel Cury AA, Derbyshire MT, Tabchoury CP. Biochemical composition and cariogenicity of dental plaque formed in the presence of sucrose or glucose and fructose. Caries Res. 2000;34(6): 491–497. 11093024 10.1159/000016629

[19] Cury JA, Francisco SB, Del Bel Cury AA, Tabchoury CP. In situ study of sucrose exposure, mutans streptococci in dental plaque and dental caries. Braz Dent J. 2001;12(2): 101–104. 11445910

[20] Mattos-Graner RO, Smith DJ, King WF, Mayer MP. Water-insoluble glucan synthesis by mutans streptococcal strains correlates with caries incidence in 12- to 30-month-old children. J Dent Res. 2000;79(6): 1371–1377. 10890715 10.1177/00220345000790060401

[21] Aires CP, Tabchoury CP, Del Bel Cury AA, Koo H, Cury JA. Effect of sucrose concentration on dental biofilm formed in situ and on enamel demineralization. Caries Res. 2006;40(1): 28–32. 16352877 10.1159/000088902

[22] Ccahuana-Vásquez RA, Tabchoury CP, Tenuta LM, Del Bel Cury AA, Vale GC, Cury JA. Effect of frequency of sucrose exposure on dental biofilm composition and enamel demineralization in the presence of fluoride. Caries Res. 2007; 41(1): 9–15. 17167254 10.1159/000096100

[23] Hefti A, Schmid R. Effect on caries incidence in rats of increasing dietary sucrose levels. Caries Res. 1979;13(5): 298–300. 288513 10.1159/000260414

[24] Imfeld TN. Identification of low caries risk dietary components. Monogr Oral Sci. 1983;11: 1–198. 6575251

[25] Jeon JG, Klein MI, Xiao J, Gregoire S, Rosalen PL, Koo H. 2009; Influences of naturally occurring agents in combination with fluoride on gene expression and structural organization of *Streptococcus mutans* in biofilms. BMC Microbiology. 2009;9: 228 10.1186/1471-2180-9-228 19863808 PMC2774857

[26] Pandit S, Cai JN, Song KY, Jeon JG. Identification of anti-biofilm components in *Withania somnifera* and their effect on virulence of *Streptococcus mutans* biofilms. J Appl Microbiol. 2015;119(2): 571–581. 10.1111/jam.12851 25976122

[27] Heydorn A, Nielsen AT, Hentzer M, Sternberg C, Givskov M, Ersbøll BK, et al Quantification of biofilm structures by the novel computer program COMSTAT. Microbiology. 2000;146(10): 2395–2407. 11021916 10.1099/00221287-146-10-2395

[28] Pandit S, Kim JE, Jung KH, Chang KW, Jeon JG. Effect of sodium fluoride on the virulence factors and composition of Streptococcus mutans biofilms. Arch Oral Biol. 2011;56(7): 643–649. 10.1016/j.archoralbio.2010.12.012 21241981

[29] Marsh PD. Dental plaque as a biofilm and a microbial community-implications for health and disease. BMC Oral Health. 2006;6(Suppl 1): S14 16934115 10.1186/1472-6831-6-S1-S14 PMC2147593

[30] Chau NP, Pandit S, Jung JE, Jeon JG. Evaluation of *Streptococcus mutans* adhesion to fluoride varnishes and subsequent change in biofilm accumulation and acidogenicity. J Dent. 2014;42(6): 726–734. 10.1016/j.jdent.2014.03.009 24694978

[31] Nobre dos Santos M, Melo dos Santos L, Francisco SB, Cury JA. Relationship among dental plaque composition, daily sugar exposure and caries in the primary dentition. Caries Res. 2002;36(5): 347–352. 12399695 10.1159/000065959

[32] Zero DT. Sugars—the arch criminal? Caries Res. 2004;38(3): 277–285. 15153701 10.1159/000077767

[33] Meldrum RJ, Brocklehurst TF, Wilson DR, Wilson PDG. The effects of cell immobilization, pH and sucrose on the growth of Listeria monocytogenes Scott A at 10°C. Food Microbiology. 2003;20: 97–103

[34] Takahashi N, Washio J, Mayanagi G. Metabolomics of supra-gingival plaque and oral bacteria. J Dent Res. 2010;89(12): 1383–1388. 10.1177/0022034510377792 20924070

